# Supplementary material for: SCN1A Variants as the Underlying Cause of Genetic Epilepsy with Febrile Seizures Plus in Two Multi-Generational Colombian Families
Source: Genes (Basel). 2022 Apr 25;13(5):754. doi: 10.3390/genes13050754 (PMC9140479; doi:10.3390/genes13050754)
Supplement: Supplementary file 1 [file genes-13-00754-s001.zip › genes-1651239 Supplementary/Supplementary File S1.pdf]

**Supplementary File S1**

We also thank the following who provided sequencing at the University of Washington Center for Mendelian Genomics (UW-CMG): Michael J. Bamshad[1,2], Suzanne M. Leal[3], and Deborah A. Nickerson[1,◊], Peter Anderson[1], Tamara J. Bacus[1], Elizabeth E. Blue[1], Katherine Brower[1], Kati J. Buckingham[1], Jessica X. Chong[1], Diana Cornejo Sánchez[3], Colleen P. Davis[1], Chayna J. Davis[1], Christian D. Frazar[1], Katherine Gomeztagle-Burgess[1], William W. Gordon[1], Martha Horike-Pyne[1], Jameson R. Hurless[1], Gail P. Jarvik[1], Eric Johanson[1], J. Thomas Kolar[1], Colby T. Marvin[1], Sean McGee[1], Daniel J. McGoldrick[1], Betselote Mekonnen[1], Patrick M. Nielsen[1], Karynne Patterson[1], Aparna Radhakrishnan[1], Matthew A. Richardson[1], Gwendolin T. Roote[1], Erica L. Ryke[1], Isabelle Schrauwen[3], Kathryn M. Shively[1], Joshua D. Smith[1], Monica Tackett[1], Gao Wang[3], Jeffrey M. Weiss[1], Marsha M. Wheeler[1], Qian Yi[1], and Xiaohong Zhang[1].

Affiliations: [1]University of Washington; [2]Seattle Children's Hospital; and [3]Columbia University.

[◊] in memoriam: January 5, 1954 to December 24, 2021. UW-CMG was funded by the National Human Genome Research Institute and the National Heart, Lung and Blood Institute grant HG006493 to Michael J. Bamshad[1,2], Suzanne M. Leal[3], Deborah A. Nickerson[1].
